# A child with autoimmune polyendocrinopathy candidiasis and ectodermal dysplasia treated with immunosuppression: a case report

**DOI:** 10.1186/1752-1947-7-44

**Published:** 2013-02-14

**Authors:** Clodagh S O’Gorman, Rayzel Shulman, Irene Lara-Corrales, Elena Pope, Margaret Marcon, Hartmut Grasemann, Rayfel Schneider, Julia Upton, Etienne B Sochett, Dror Koltin, Eyal Cohen

**Affiliations:** 1 Divisions of Endocrinology, The Hospital for Sick Children, Toronto, Canada; 2 Department of Pediatrics, The Hospital for Sick Children, Toronto, Canada; 3 Divisions of Dermatology, The Hospital for Sick Children, Toronto, Canada; 4 Divisions of Gastroenterology, Hepatology and Nutrition, The Hospital for Sick Children, Toronto, Canada; 5 Divisions of Respiratory Medicine, The Hospital for Sick Children, Toronto, Canada; 6 Divisions of Rheumatology, The Hospital for Sick Children, Toronto, Canada; 7 Divisions of Immunology, The Hospital for Sick Children, Toronto, Canada; 8 Divisions of Pediatric Medicine, The Hospital for Sick Children, Toronto, Canada; 9 University of Toronto, Toronto, Canada; 10 Current affiliation is Department of Paediatrics, Graduate Entry Medical School, University of Limerick, and University Hospital, Limerick, Ireland; 11 Graduate Entry Medical School, University of Limerick, Limerick, Ireland

**Keywords:** APECED (autoimmune polyendocrinopathy-candidiasis-ectodermal dysplasia), APS (autoimmune polyendocrinopathy syndrome), Autoimmunity, Immunosuppression, Endocrinopathies

## Abstract

**Introduction:**

Common features of autoimmune polyendocrinopathy-candidiasis-ectodermal dysplasia include candidiasis, hypoparathyroidism and hypoadrenalism. The initial manifestation of autoimmune polyendocrinopathy-candidiasis-ectodermal dysplasia may be autoimmune hepatitis, keratoconjunctivitis, frequent fever with or without a rash, chronic diarrhea, or different combinations of these with or without oral candidiasis.

**Case presentation:**

We discuss a profoundly affected 2.9-year-old Caucasian girl of Western European descent with a dramatic response to immunosuppression (initially azathioprine and oral steroids, and then subsequently mycophenolate mofetil monotherapy). At four years of follow-up, her response to mycophenolate mofetil is excellent.

**Conclusion:**

The clinical features of autoimmune polyendocrinopathy-candidiasis-ectodermal dysplasia may continue for years before some of the more common components appear. In such cases, it may be life-saving to diagnose autoimmune polyendocrinopathy-candidiasis-ectodermal dysplasia and commence therapy with immunosuppressive agents. The response of our patient to immunosuppression with mycophenolate mofetil has been dramatic. It is possible that other patients with this condition might also benefit from immunosuppression.

## Introduction

The autoimmune regulator (*AIRE*) gene, on chromosome 21, expresses self-antigens in the thymus and mediates negative selection of self-reactive T-cells [1]. Autoimmune polyendocrinopathy-candidiasis-ectodermal dysplasia (APECED), or autoimmune polyendocrinopathy syndrome type I, occurs due to loss of function of *AIRE*, with resultant loss of thymic tolerance to self-antigens [1].

Common features of APECED include candidiasis, hypoparathyroidism and adrenal insufficiency [2]. The initial manifestation of APECED may be autoimmune hepatitis, keratoconjunctivitis, frequent fever with or without a rash, chronic diarrhea, or different combinations of these with or without oral candidiasis. This clinical picture may continue for years before the appearance of the more common components [2]. In such cases, it may be life-saving to diagnose APECED and commence therapy with immunosuppressive agents. The literature on therapeutic options for severe manifestations of this rare condition is scarce. Some data suggest that a younger age at presentation is associated with increased disease severity, although the authors do not specify how young [2]. We discuss a profoundly affected young patient with a dramatic response to immunosuppression using mycophenolate mofetil (MMF).

## Case presentation

**Figure 1:**
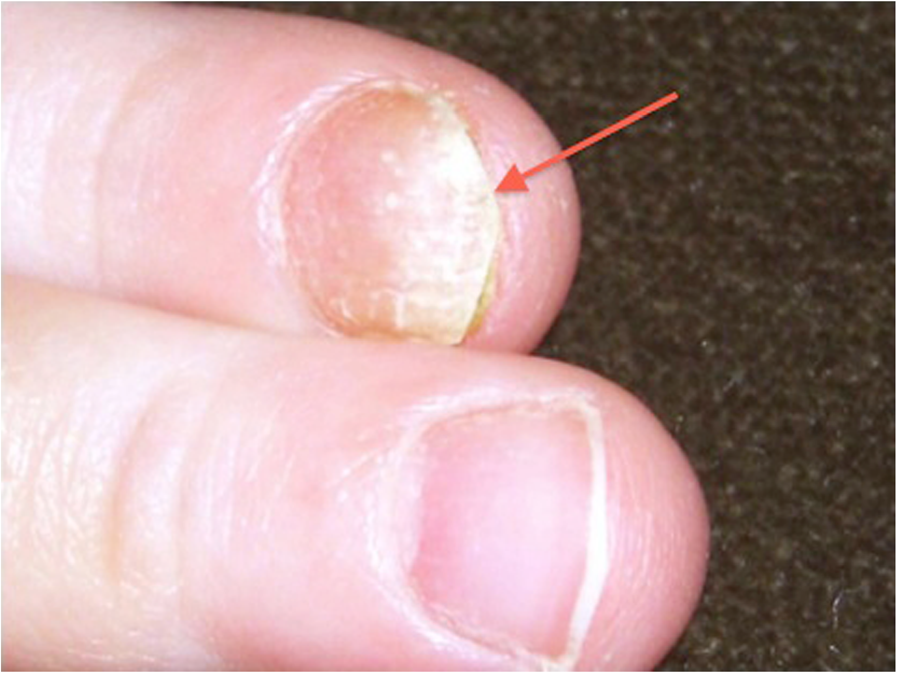
Mucocutaneous candidiasis (red arrow).

**Figure 2:**
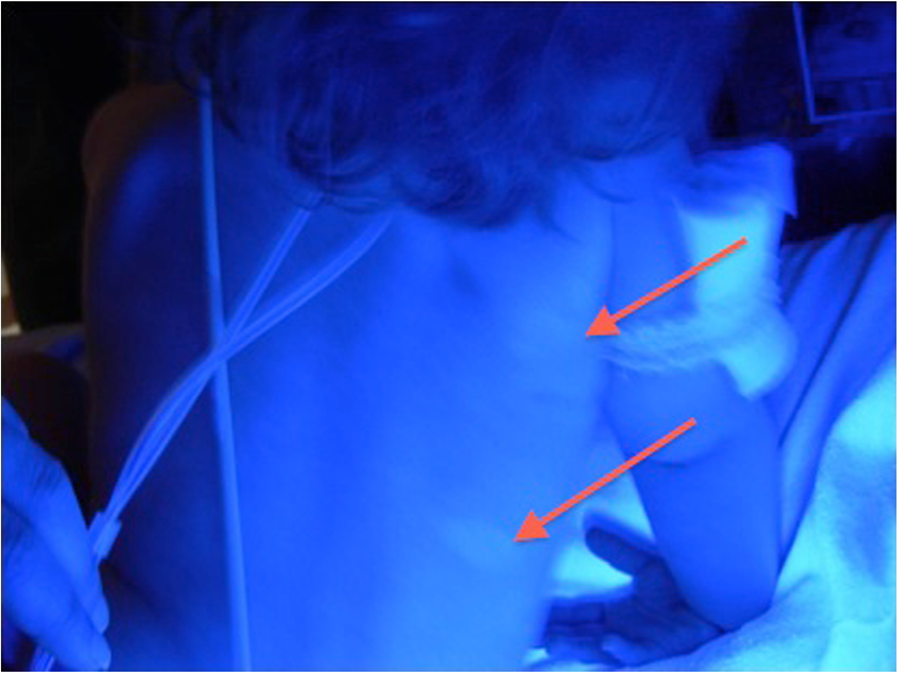
Vitiligo (red arrows) (seen under Wood’s lamp).

**Figure 3:**
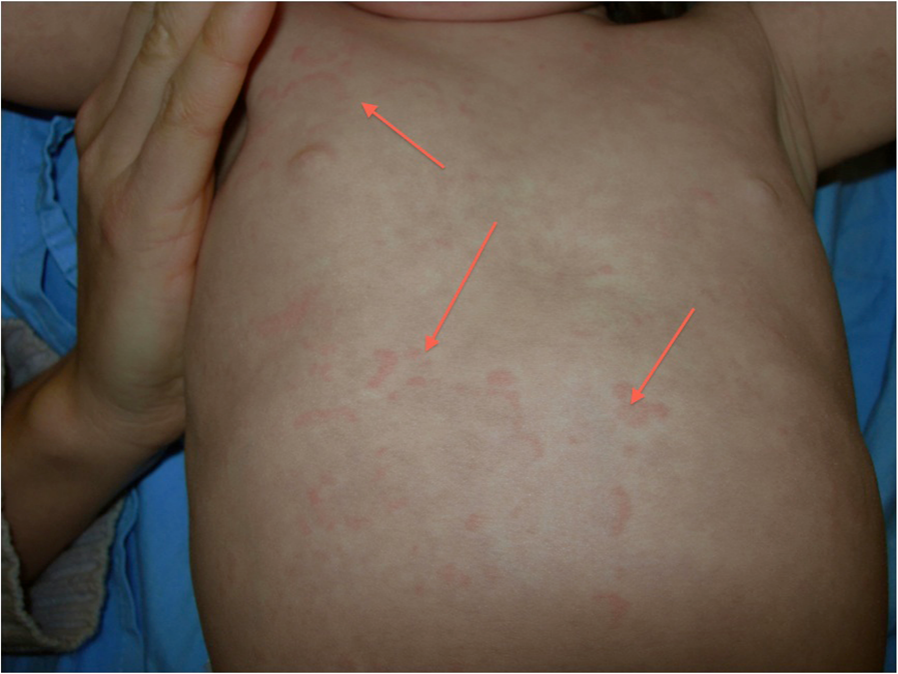
Urticaria-like eruption (red arrows).

A 2-year-old girl of non-consanguineous Caucasian parents of Western European descent, developed the following clinical features over 12 months: hypoparathyroidism with hypocalcemia and borderline high serum phosphate (parathyroid hormone 20ng/L, normal 10 to 65; total calcium 1.98mmol/L, normal 2.2 to 2.6; phosphate 2.1mmol/L, normal 1.6 to 2.1); mucocutaneous candidiasis (Figure 1); keratoconjunctivitis; sialoadenitis; vitiligo (Figure 2); urticaria-like rash (Figure 3); and alopecia. At age 2.9 years, she required intensive care admission and ventilation for aspiration pneumonia and significant respiratory compromise. During this admission, severe hypoparathyroidism with significant hypocalcemia was managed with high doses of intravenous and subsequently oral calcium and calcitriol. APECED was confirmed following identification of a homozygous 13 base pair deletion on *AIRE* exon 8; both parents were heterozygous carriers.

Previously, she had been investigated and treated for several episodes of aspiration pneumonia, frequent diarrhea, intermittent urticaria-like eruptions (Figure 3) with lymphocytic vasculitis on biopsy and frequent fevers of unknown origin. Between diarrheal illnesses, she had severe constipation, including a partial small bowel obstruction at 1.3 years old.

Laboratory evaluation revealed: normal thyroid function tests; positive anti-thyroid peroxidase antibodies; normal glucocorticoid function following adrenal stimulation; positive anti-adrenal and anti-21-hydroxylase antibodies; normal electrolytes and mineralocorticoid function; intercurrent stress hyperglycemia; positive anti-glutamic acid decarboxylase antibodies but negative islet cell and anti-insulin antibodies. While ventilated, *Candida albicans* was cultured from her endotracheal tube; *C. albicans* and organic food material were isolated following closed lung biopsy.

Her diarrhea worsened, and became associated with deterioration of her hypoparathyroidism and hypocalcemia requiring prolonged parenteral calcium. Hypocalcemia was marked after discontinuing parenteral calcium for even a few hours. She then developed mineralocorticoid insufficiency, worsening sicca symptoms, and recurrent life-threatening respiratory tract infections.

After seven months of hospitalization, immunosuppression was commenced, first using high dose oral prednisone (two mg/kg), then azathioprine (1.5mg/kg). Calcium and diarrhea stabilized somewhat. However, repeated attempts to wean maintenance steroids failed due to exacerbations of diarrhea and hypocalcemia. Therefore, immunosuppression was changed to MMF monotherapy (900mg/m^2^). Prednisone was weaned successfully to physiological maintenance doses, and continued at these doses.

**Figure 4:**
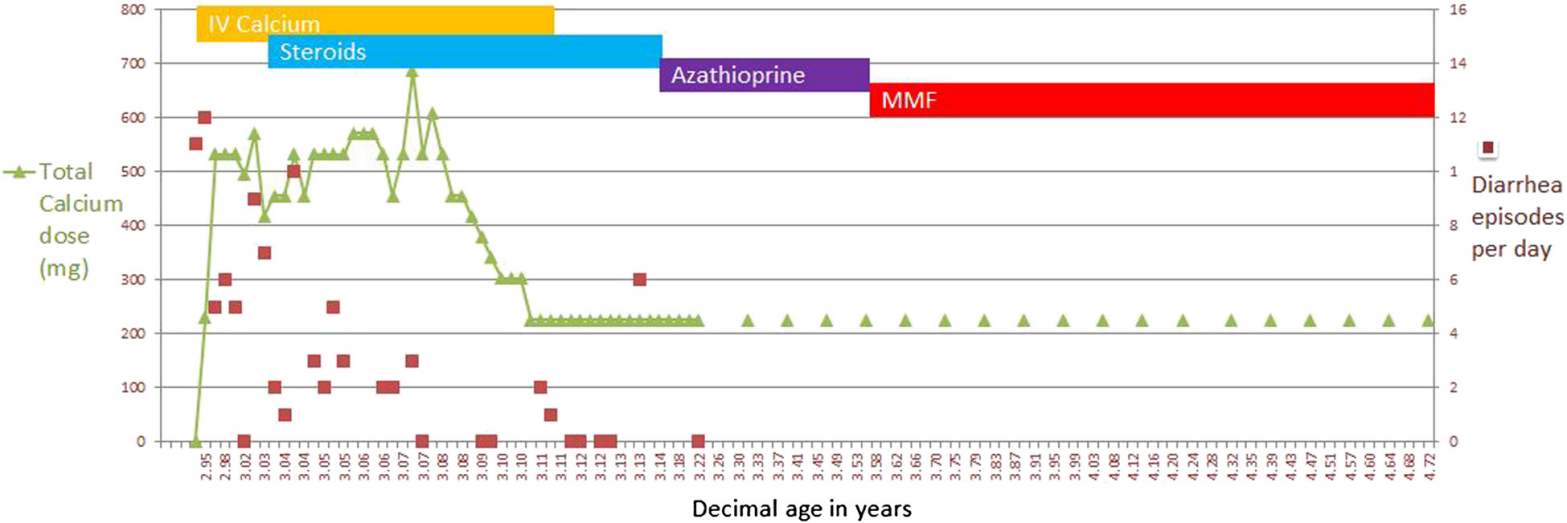
**Graph of calcium intake, serum calcium and diarrhea over time, including response to immunosuppression.** Ca; Calcium.

Her clinical response to immunosuppression was dramatic. Intravenous calcium was discontinued after one week; diarrhea began to resolve (Figure 4). Within one month of starting MMF, she was discharged home on oral calcium and calcitriol supplements. Linear growth, salivation, lacrimation and repigmentation of her eyelashes improved.

She is now seven years old, height 110.7cm (3^rd^ centile) and weight 19.2kg (16^th^ centile). She still has frequent episodes of fever, although not consistently related to infections. She has had documented throat and lower respiratory tract infections, but these have responded well to antibiotics without further support. Her lung function testing is normal. MMF was titrated to achieve target area under the curve of 40 to 60mg/hour/L. Notably, when MMF is held for even two to three days (for example during febrile illnesses), despite doubling or tripling steroids for stress dosing, diarrhea deteriorates, she becomes hypocalcemic, requiring increased calcium and calcitriol supplementation (Figure 4).

## Discussion

This case highlights a dramatic response of a young patient with APECED to immunosuppression with MMF therapy as a steroid-sparing immunosuppressant, despite an initial poor response to azathioprine. To the best of our knowledge, our patient with APECED is the youngest treated with immunosuppression to date; see Table 1 for manuscripts describing immunosuppression for patients with APECED. The initial clinical manifestations of APECED may be autoimmune hepatitis, keratoconjunctivitis, frequent fever with or without a rash, chronic diarrhea, or different combinations of these with or without oral candidiasis [2]. This clinical picture may continue for years before some of the more common components appear. In such cases, it may be life-saving to diagnose APECED and commence therapy with immunosuppressive agents [2]. For atypical cases, a simple diagnostic test for APECED (autoantibodies against interferon omega) is available [3].

**Table 1:** Summary of case reports of use of immunosuppression in patients with APECED

***Year of publication***	***Authors of manuscript***	***Age of patient at initiation of immunosuppression (years)***	***Immunosuppression medications used***
1997	Padeh *et al*. [4]	15	Methylprednisolone for induction; methotrexate for maintenance
1999	Ward *et al*. [5]	13	Cyclosporin A
2006	Perheentupa [2]	Various ages	Prednisone and azathioprine combination
2006	Ulinski *et al*. [7]	6	Prednisone and azathioprine; then plus cyclosporin A; then changed to prednisone, MMF and tacrolimus
2007	Bakrac *et al*. [8]	33	Prednisone and cyclosporin A; then MMF
This manuscript	O’Gorman *et al*.	2.9	Methylprednisolone; then prednisone and azathioprine; then MMF

MMF; mycophenolate mofetil.

In a 13-year-old patient diagnosed with APECED at age 2.5 years, the successful use of steroids, followed by three years of methotrexate maintenance has been described [4]. When immunosuppression was started, she had mucocutaneous candidiasis, alopecia, hypoparathyroidism, adrenal insufficiency and pancreatic insufficiency [4]. Another 13-year-old patient with APECED had improvements in diarrhea and growth following six months of immunosuppression [5]. This patient’s disease is now controlled with MMF (Personal communication, Cheri Deal, 2009). In 2002, Perheentupa discussed prednisone and azathioprine for chronic hepatitis in a patient with APECED and referred to glucocorticoids and either azathioprine or methotrexate in several patients with diarrhea [6]. Immunosuppression with prednisone and azathioprine, and subsequently with cyclosporin A, was reported following renal transplantation for chronic interstitial nephritis in a patient with APECED. Immunosuppression was unsuccessful with azathioprine but successful with cyclosporin A [7]. A patient with APECED with pure red cell aplasia responding well to MMF has also been described [8].

Two cautions regarding the use of immunosuppression for patients with APECED are reported [6]: increased risk of candidiasis-related malignancies, such as oral malignancies; and increased risk of the progression from localized to systemic candidiasis. Six patients with APECED with oral or esophageal squamous cell carcinomas have been described; the youngest was 29 years old and immunosuppressed for six years; the others were not immunosuppressed [6]. It is unclear if candida-related malignancies in APECED are related to APECED or to its treatments [9]. It is also important to note that immunosuppression for a patient with APECED is really a decision tailored to the specific details of an individual patient. In this case, significant consideration and discussion among many health professionals and the patient’s family occurred before immunosuppression was started. Furthermore, any decision to initiate immunosuppression should be accompanied by a definite plan for monitoring the response to this new therapy.

After four years of follow-up, we have not observed significant side-effects of immunosuppression in our young patient. When she presents with febrile or infectious illnesses, MMF is temporarily discontinued but, within 2–3 days, diarrhea increases and she develops hypocalcemia, requiring increased enteral calcium and calcitriol supplementation. For our patient, inflammatory markers, diarrhea, required doses of calcium supplementation and weight gain are good markers of disease activity. Abnormal swallowing and silent aspirations are not recognized features of APECED and it is possible that our patient’s respiratory symptoms may represent new autoimmune-related APECED manifestations or another co-incident disease process. Chronic diarrhea is a recognized feature of APECED [6]. Our patient’s diarrhea may represent autoimmune gastrointestinal disease, but this was not biopsy proven before immunosuppression. Obstipation is a recognized manifestation [10] and our patient has not had bowel obstructions since MMF was commenced.

## Conclusions

Immunosuppression is well tolerated and has resulted in dramatic improvement in our young patient with severe manifestations of APECED. We suggest that early initiation of immunosuppression, with appropriate monitoring, is an acceptable therapy for patients severely affected with APECED and that failure of one immunosuppressive agent does not infer failure of others.

## Consent

Written informed consent was obtained from the parents of this patient (minor) for publication of this case report and accompanying images. A copy of the written consent is available for review by the Editor-in-Chief of the Journal of Medical Case Reports.

## Abbreviations

AIRE: Autoimmune regulator; APECED: Autoimmune polyendocrinopathy-candidiasis-ectodermal dysplasia; MMF: Mycophenolate mofetil.

## Competing interests

The authors declare that they have no competing interests.

## Authors’ contributions

CSOG conceived the case report, collected the data, interpreted the data, and was a major contributor in writing the manuscript including writing the first draft. RSh participated in collection and interpretation of data, and contributed to writing the manuscript. ILC participated in collection and interpretation of data and contributed to writing the manuscript. EP, MM, HG, RSch, JU, and EBS participated in data interpretation and contributed to writing the manuscript. DK participated in collection and interpretation of data, and was a major contributor in writing the manuscript. EC conceived the case report, participated in data interpretation, was a major contributor in writing the manuscript. All authors read and approved the final manuscript.
